# Pathway of peritoneal carcinomatosis maybe hematogenous metastasis rather than peritoneal seeding

**DOI:** 10.18632/oncotarget.14607

**Published:** 2017-01-12

**Authors:** Wei Ge, Gang Chen, Xiang-Shan Fan

**Affiliations:** ^1^ Department of general surgery, Nanjing Drum Tower Hospital, The Affiliated Hospital of Nanjing University Medical School, Nanjing, Jiangsu Province, P. R. China; ^2^ Department of Pathology, Nanjing Drum Tower Hospital, The Affiliated Hospital of Nanjing University Medical School, Nanjing, Jiangsu Province, P. R. China

**Keywords:** peritoneal carcinomatosis, seeding, hematogenous metastasis, GC, colon cancer

## Abstract

**Goals:**

This study aimed to summarize the clinicopathological data of the cases of gastric cancer or colon cancer with regular metastasis in the mesentery of small intestine and explore the pathway of peritoneal carcinomatosis.

**Materials and methods:**

We retrospectively analyzed 5 cases of gastric cancer and 3 cases of colon cancer with regular metastasis in the mesentery of the small intestine from January 2014 to June 2016, including clinical information, gross manifestations during operation, treatment, and pathology.

**Results:**

The clinical characteristics of all 8 cases were fully collected. The symptoms were various without specificity. All patients were found to present with metastasis in peritoneum during operation and the metastatic lesions arranged along the blood vessels orderly. The metastatic lesions of all studied patients were proved to be malignant carcinoma histopathologically, the same as the original tumor. Tumor emboli were seen in the vessel and invasive neoplastic foci was also seen in the vascular wall.

**Conclusions:**

The traditional view that peritoneal carcinomatosis is due to seeding has no sufficient basis. Hematogenous metastasis maybe the real way of peritoneal carcinomatosis combined with clinical presentation.

## INTRODUCTION

The outcome of gastric cancer (GC) remains poor despite of the use of interdisciplinary approaches to treatment and palliation [1]. Curative resection (R0) is still the mainstay treatment for this disease [2]. However, even after R0 resection plus adjuvant therapy, the 5-year survival rate for GC has not improved substantially in decades and the recurrence rate is still high [3]. Peritoneal carcinomatosis is the most frequent pattern of recurrence in patients with GC [4]. In the past, peritoneal carcinomatosis has been regarded as a terminal disease, especially when the primary tumor was GC, and most oncologists would regard it as a condition only to be palliated. According to the traditional point of view, peritoneal carcinomatosis is considered to be caused by free cancer cells exfoliated from serosa-invasive tumors. However, the pathogenesis has not been fully covered at present. Of course, there are other viewpoints against this conclusion.

In this study, we summarized the clinical data of some cases of GC or colon cancer with regular metastasis in the mesentery of small intestine. We imaged that the pathway of peritoneal carcinomatosis maybe hematogenous metastasis rather than seeding.

## MATERIALS AND METHODS

We reviewed 5 cases of GC and 3 cases of colon cancer with regular metastasis in the mesentery of the small intestine from January 2014 to June 2016. This study was approved by IRB of Nanjing Drum Tower Hospital, the affiliated hospital of Nanjing University Medical School. The informed consents for participation in the study were obtained from all participants. We recorded the patients^,^ age, gender, clinical symptoms, diagnosis, treatment, pathological examination, and follow-up information. We focused on analyzing the histopathological examination of surgical specimens using standard hematoxylin and eosin staining (H&E), as well as immunohistochemical techniques.

## Results

### Baseline characteristics

**Figure 1:**
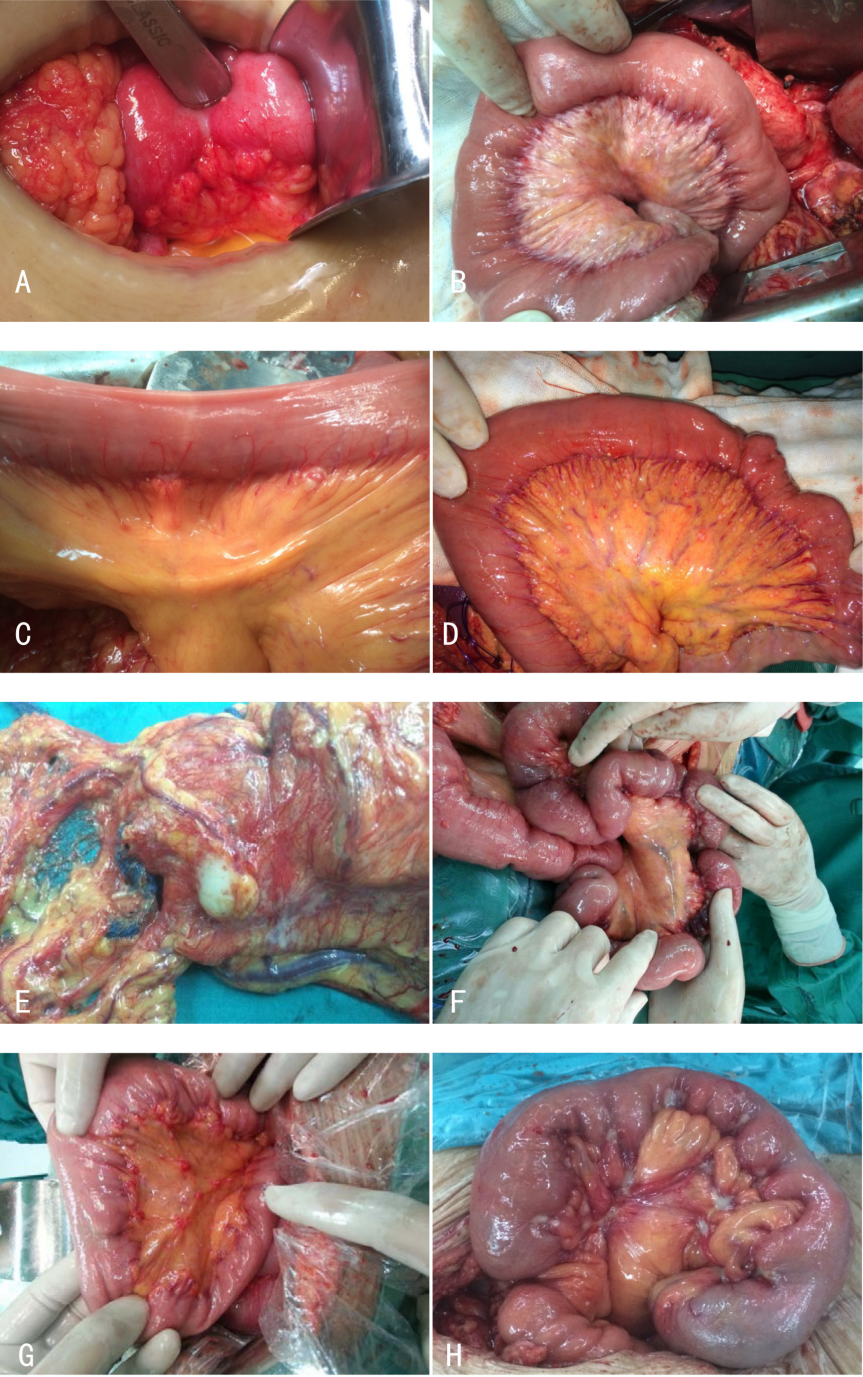
**A**. GC with metastasis in mesentery of the small intestine in a 60-year-old male patient. **B**. GC with metastasis in mesentery of the small intestine and colon in a 44-year-old male patient. **C**. GC with metastasis in mesentery of the small intestine in a 70-year-old male patient. **D**. GC with metastasis in abdominal wall and mesentery of the small intestine in a 63-year-old male patient. **E**. Colon cancer with metastasis in mesocolon in a 42-year-old female patient. **F**. Colon cancer with metastasis in mesentery of the small intestine in a 58-year-old female patient. **G**. Colon cancer with metastasis in abdominal wall and mesentery of the small intestine in a 80-year-old male patient. **H**. GC with metastasis in mesentery of the small intestine in a 62-year-old male patient.

The linical characteristics of all of 8 cases were fully collected (6 men and 2 women, age ranging from 42 to 80 years old, with the mean age of 59.9 years). The clinical data were summarized in Table 1. The symptoms were various with no specificity, such as abdominal pain, abdominal distention, ileus and so on. All patients underwent surgical operative treatment. Metastasis in peritoneum was found in all 8 patients. The metastatic lesions arranged along the blood vessels regularly (Figure 1).

**Table 1:** Clinical data of the studied patients

No	Sex	Age	Diagnosis	Operation	Metastasis site
1	Male	60	Adenocarcinoma	Exploratory laparotomy	Mesentery of the small intestine
2	Male	44	Adenocarcinoma	Palliative distal gastrectomy	Mesentery of the small intestine and colon
3	Male	70	Adenocarcinoma	Palliative total gastrectomy	Mesentery of the small intestine and colon
4	Male	63	Adenocarcinoma	Exploratory laparotomy	Abdominal wall and mesentery of the small intestine
5	Female	42	Adenocarcinoma	Radical correction of colon transversum carcinoma	Mesocolon
6	Female	58	Adenocarcinoma	Lesion biopsy and jejunostomy	Mesentery of the small intestine
7	Male	80	Adenocarcinoma	Lesion biopsy and short circuit operation	Abdominal wall and mesentery of the small intestine
8	Male	62	Adenocarcinoma	Small intestine local excision and jejunostomy	Mesentery of the small intestine

### Pathological findings

**Figure 2:**
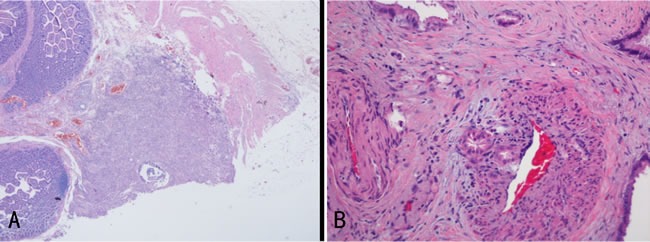
**A**. Pathological finding of metastatic lesion by hematoxylin and eosin staining: poorly differentiated adenocarcinoma originating from stomach infiltrated the submucosa of the small intestine in this case. **B**. Invasive neoplastci foci were seen in the vascular wall.

The metastatic lesions of all studied patients were proved to be adenocarcinoma histopathologically, the same as the primary tumor (Figure 2A). Tumor emboli were seen in the vessel and the invasive neoplastic foci was also seen in the vascular wall (Figure 2B). The primary tumors of seven patients were removed for pathological examination. Cases presenting nerve invasion were observated in all of the seven patients, and vessel invasion in five patients. Of all the eight patients, seven suffered from serosa infiltration. All these were summarized in Table 2.

**Table 2:** Pathological findings of the studied patients

No	Differentiated degree	Nerve invasion	Vessel invasion	Metastatic/cleaned Lymph nodes	Serous infiltration
1	NA	NA	NA	NA	Yes
2	Poorly	Yes	No	6/37	No
3	Poorly	Yes	Yes	9/12	Yes
4	Poorly	Yes	Yes	NA	Yes
5	Moderately	Yes	No	0//21	Yes
6	Moderately	Yes	Yes	NA	Yes
7	Well	Yes	Yes	NA	Yes
8	Poorly	Yes	Yes	18/19	Yes

Abbreviation: NA=data not available

## DISCUSSION

It was once believed that peritoneal carcinomatosis of gastrointestinal cancer was seeding metastasis. The theory of” seed and soil” proposed by Steven Paget in 1989 was generally regarded as pathogenetic mechanism of seeding metastasis. Peritoneal carcinomatosis should be irregular lesions according to this theory. However, the metastatic lesions distributed regularly along the vessel and proved to be the same to the original tumor in our study. Therefore, we ruled out the possibility of seeding metastasis. Besides, the metastatic lesion was cancer nodules rather than lymph node. So we deduced that the Pathway of metastasis was hematogenous in our study. We boldly put forward the idea that hematogenous metastasis was the real way of peritoneal carcinomatosis in combination with related literature.

Some patients were found to have peritoneal carcinomatosis when the tumor had not invaded the serosa. In our study, one patient without serosal invasion was also discovered to peritoneal carcinomatosis during operation. If the peritoneal carcinomatosis was due to seeding, where did the exfoliated cancer cells come from. Previously, Ishida et al. raised the “second metastasis” theory that tumor metastasized to lymph nodes, passed through the capsule to the abdominal cavity and seeded [5]. For peritoneal dissemination in non-serosal- invasive carcinoma, Marutsuka et al. postulated that lymph node dissection during operation opened lymphatic channels and spread viable cancer cells into the abdominal cavity [6]. However, all these theories firmly supported that the reason for peritoneal carcinomatosis was seeding rather than hematogenous metastasis. In fact, since the cancer cells could transfer to liver, lung, bone, brain and so on. Why can't they transfer to the peritoneum.

Most scholars agree that cancers outside of the abdomen and hematological malignancy transfer to the peritoneum through blood or lymph vessel. However, everyone agrees that intraperitoneal tumor transfer to peritoneum by seeding. The clear and definite evidence has not been fully established thus far. For serosal- invasive carcinoma, exfoliated cancer cells found in ascetic fluid or peritoneal lavage fluid, together with metastasis in the peritoneum, lead to the viewpoint that peritoneal carcinomatosis was seeding dissemination. In fact, these two phenomena had no inevitable causation. The exfoliated cancer cells definitely would not grow on the peritoneum and form metastases.

Peritoneal is tough and thick serous tissue, made up of three layers. The outermost layer is consisting of simple squamous mesothelial cells, which is a mechanical defence barrier, avoiding the exposure of the subcutaneous tissue and microbial attack. Besides, it could create many cytokines to participate in peritoneal repair and host defense. The innermost layer was connective tissue, in which many different diameters of blood and lymphatic vessels were distributed. Intermediate layer is basement membrane to divide the outermost and innermost layer. So the innermost layer is a good environment for the growth of tumors. Because of peritoneal mechanical barrier and host defense function, it is difficult for the exfoliated cancer cells to engraft and seed on the peritoneum. Therefore, the metastatic lesion is formed through subperitoneal microvascular or lymphatic vessels, which seems to be more in line with the clinical phenomenon.

If the peritoneal carcinomatosis was really due to tumor seeding, a large number of cancer cells would fall off in serosal- invasive carcinoma and the peritoneal carcinomatosis rate would be very high. However, the peritoneal carcinomatosis rate was just 10% in advanced GC. Koppe et al. reported that peritoneal carcinomatosis was encountered in approximately 7% of patients with colorectal cancer at primary surgery, in approximately 4%-19% of patients during follow-up after curative surgery [7]. Peritoneal carcinomatosis was also reported in patients with cancer of liver, pancreas, and bile duct with low incidence. In contrast, the rate in patients with ovarian cancer was high. Rose PG et al. found that peritoneal carcinomatosis rate was approximately 85%-100% by autopsy [8]. The difference was so large that the old theory of peritoneal seeding could not explain it. We speculate that tumors initiating different organs have different molecular biological characteristics and tumor stem cells have their own “prefer” target organs. So some tumors are easy to transfer to liver, and some are easy to transfer to peritoneum.

Krukenberg tumor was metastatic ovarian tumor derived from gastrointestinal malignant tumor, as well as appendix and pancreas [9, 10]. It has long been recognized that cancer cells invaded serosa and seeded on the ovary, forming Krukenberg tumor. Ascites was the common symptom of patient with Krukenberg tumor and cancer cells found in ascites was the direct evidence for seeding metastasis [11]. However, many scholars argued this metastatic route. Because the serosa of Krukenberg tumor was smooth and complete and the tumor growth in ovarian serous membrane, mainly involved ovarian medulla. Seeding metastases should present as invasive growth outside the ovary.

In 2003, Japanese scholar reported a case of Krukenberg tumor in a patient with early GC confined to the mucosa, denying the seeding metastasis further [12]. More researchers support the hematogenous metastatic route, as ovary is rich in blood vessels and cancer embolus are usually found. Krukenberg tumor often occurs in premenopausal women. Patients with Krukenberg tumor are younger than those with primary ovarian cancer. The reason is that functional active ovary is rich in blood supply and hormone, easy to attract the cancer cells, providing a favorable environment for the cancer cells to engraft and grow [13, 14]. So the hematogenous metastasis is an important pathway of peritoneal carcinomatosis.

Mesangial metastatic carcinoma nodules were also seen in patients with colorectal cancer and there was normal tissue between primary tumor and metastatic nodule. This phenomenon was ever also thought to be caused by tumor cells seeding. However, it was difficult for the exfoliated cancer cells to pass through in the mesentery and seeded. So we thought that all these were due to hematogenous metastasis. Besides, skin metastasis from malignant tumor was also common. Although the pathogenesis was unclear, most scholars supported hematogenous metastasis. We concluded that hematogenous metastasis was not only the way to metastasize to the distant organs of abdominal malignancy but also the way to the peritoneum.

Studies have found that patients with malignant tumor released at least one hundred million of cancer cells once a day into the blood and became circulating tumor cells (CTCs). In 2005, Pachmann defined CTCs as circulating tumor cells released from solid tumor or metastases spontaneously or by operation of diagnosis and treatment [15]. CTCs could not only spread to other organs forming metastases, but also home to the original site and accelerate the growth of tumor under the action of the tumor chemokine. Studies showed that survival of patients with positive CTCs was significantly shorter than that of patients with negative CTCs. [16, 17] Smerage et al. tested the CTCs in patients with breast cancer and found that increased CTCs was associated with poor prognosis [18].

The majority of circulating tumor cells appeared to be destroyed. Those that persist may acquire ability to metastasize and once inside the target organ may undergo Mesenchymal-Epithelial Transition(MET), proliferate and if the environment is conducive, the disseminated cells may grow to establish a new tumor thus competing the metastatic process. These CTCs has all the characteristics of the stem cells: capability of self-renewal, unlimited proliferation potential, multiline differentiation, formation of new adult cells, and asymmetric division. For these properties, they are called cancer stem cells (CSCs) [19].

There were evidences to verify the existence of CSC. Leukemia cancer cells divided and amplified like stem cells [20]. Multipotential stem cells of human teratoma could differentiate into all kinds of organizations, such as muscle and bone [21]. We separated CSC with self-renewal and multi-directional differentiation potential from tumors of the human central nervous system [22, 23]. We also confirmed that the breast stem cells were the source of the breast cancer [24]. So we think that tumor cell homing phenomenon was the real mechanism of peritoneal carcinomatosis.

In our study, peritoneal carcinomatosis distributed along the blood vessel regularly, which was the direct evidence that peritoneal carcinomatosis was via hematogenous dissemination. Digestive tract malignancies metastasize to small mesenteric and parietal peritoneum through hematogenous dissemination. The homing effect of tumor cells may be the important mechanism of the formation of peritoneal carcinomatosis.

## PROSPECT

Some scholars put forward that the peritoneal lavage cytology test to find cancer cells was an important method to detect the peritoneal carcinomatosis. Hyperthermic intraperitoneal chemotherapy could reduce the metastasis rate and improve the prognosis. Costa and Glehen had confirmed that surgery combined with hyperthermic intraperitoneal chemotherapy could prolong survival period [25, 26].

We concluded the reason for hyperthermic intraperitoneal chemotherapy improving prognosis was that chemotherapy drugs were absorbed into the blood and removed the CTCs and CSCs.

Many scholars tried to filter CTCs by cell strainers and had made breakthrough progress. In 1997, Kongs-gaard et al. filtered CTCs by leukocyte depletion filter [27]. Besides, nucleated cells purifier, chip filter and so on could improve the filtration efficiency [28, 29]. These methods could reduce tumor metastasis and recurrence, worth further study.

## CONCLUSION

The traditional view that peritoneal carcinomatosis is due to seeding has no sufficient basis. Hematogenous metastasis maybe the real way of peritoneal carcinomatosis combined with clinical presentation. However, more evidences are necessary in this study.
